# A Precision Medicine Agenda in Traumatic Brain Injury

**DOI:** 10.3389/fphar.2022.713100

**Published:** 2022-03-16

**Authors:** Jovany Cruz Navarro, Lucido L. Ponce Mejia, Claudia Robertson

**Affiliations:** ^1^ Departments of Anesthesiology and Neurosurgery, Baylor College of Medicine, Houston, TX, United States; ^2^ Departments of Neurosurgery and Neurology, LSU Health Science Center, New Orleans, LA, United States; ^3^ Department of Neurosurgery, Baylor College of Medicine, Houston, TX, United States

**Keywords:** precision medicine, traumatic brain injury, biomarkers, neuromonitoring, genomics and epigenomics

## Abstract

Traumatic brain injury remains a leading cause of death and disability across the globe. Substantial uncertainty in outcome prediction continues to be the rule notwithstanding the existing prediction models. Additionally, despite very promising preclinical data, randomized clinical trials (RCTs) of neuroprotective strategies in moderate and severe TBI have failed to demonstrate significant treatment effects. Better predictive models are needed, as the existing validated ones are more useful in prognosticating poor outcome and do not include biomarkers, genomics, proteonomics, metabolomics, etc. Invasive neuromonitoring long believed to be a “game changer” in the care of TBI patients have shown mixed results, and the level of evidence to support its widespread use remains insufficient. This is due in part to the extremely heterogenous nature of the disease regarding its etiology, pathology and severity. Currently, the diagnosis of traumatic brain injury (TBI) in the acute setting is centered on neurological examination and neuroimaging tools such as CT scanning and MRI, and its treatment has been largely confronted using a “one-size-fits-all” approach, that has left us with many unanswered questions. Precision medicine is an innovative approach for TBI treatment that considers individual variability in genes, environment, and lifestyle and has expanded across the medical fields. In this article, we briefly explore the field of precision medicine in TBI including biomarkers for therapeutic decision-making, multimodal neuromonitoring, and genomics.

## Introduction and Background

Traumatic brain injury (TBI) remains a major disease and it is of public health interest across the globe. Its incidence and mechanism of trauma vary by region however, it has been reported in the Global Burden of Disease (GBD) study to be around 369 cases per 100,000 people worldwide (GBD 2016 Traumatic Brain Injury and Spinal Cord Injury Collaborators, 2019). The GBD study estimates a TBI incidence of 1.11 million and prevalence of 2.35 million in the United States in 2016 (GBD 2016 Traumatic Brain Injury and Spinal Cord Injury Collaborators, 2019), with an incidence rate of 333 per 100,000 (a 3.3 percent reduction from 1990), whereas the prevalence rate was 605 per 100,000 (5.7 percent reduction compared with 1990) (GBD 2016 Traumatic Brain Injury and Spinal Cord Injury Collaborators, 2019). The Centers for Disease Control and Prevention (CDC) estimates that approximately 227,000 TBI related hospitalizations occurred in 2016 and counts decreased to almost 224,00 in 2017; with unintentional falls and motor vehicle crashes accounting for the majority of these hospitalizations (Prevention, 2017). Approximately 60,000 TBI-related deaths occurred in 2016, and deaths increased to over 61,000 in 2019 (Prevention, 2020). Suicide and unintentional falls were the most common mechanisms of injury contributing to a TBI-related death. Suicide accounted for 33.8% of all TBI-related deaths in 2016 and 34.7% in 2017. Unintentional falls accounted for approximately 28% of all TBI-related deaths in both years (Prevention, 2017). TBI remains as the most common cause of death and disability in young adults worldwide (Ghajar, 2000). Substantial uncertainty in outcome prediction continues to be the rule despite the existing prediction models. This is in part due to traumatic brain injury being a heterogeneous disease regarding its etiology, pathology and severity. A number of prediction models have been developed to establish prognosis in victims of TBI. In the U.S. the *All of Us* Research Program (formerly known as the Precision Medicine Initiative Cohort Program) will be a participant-engaged, data-driven enterprise supporting research at the intersection of lifestyle, environment, and genetics to produce new knowledge with the goal of developing more effective ways to prolong health and treat disease. TBI research must rely in such initiatives for the advancement in the diagnosis, prognosis and management of head injury (Table 1).

**TABLE 1:** Different components of the multimodal approach used in precision medicine.

	Definition	Current technology available for clinical use and clinical end-points
Genomics and Proteomics	Genome-wide association studies identify genes that are associated with susceptibility to a disease or affect its outcome (NINDS Stroke Genetics Network (SiGN)International Stroke Genetics Consortium (ISGC), 2016).	—
Metabolome	Metabolomics identifies changes in bioenergetic metabolism, changes in metabolite concentration, and any alteration in normal processes	—
Endophenotypes		
Epigenetics	Epigenetics refers to reversible modifications in gene expression related to attachment of certain compounds to chromatin	—
	Enviromental factors may affect these compounds which tend to be inheritable	
Biomarkers	Several biomarkers can assist in TBI diagnosis and prognostication including neuron specific enolase, microtubule-associated protein, protein S100 B, glial fibrillary acidic protein, microRNAs, and tau protein among others	FDA approved Brain Trauma Indicator and i-STAT Alinity TBI plasma test to measure UCH-L1 and glial fibrillary acidic protein (GFAP) for determination of clinical need of a CT after mild TBI.
Predictive modeling	Uses data mining, statistics and probability, modeling, machine learning and artificial intelligence to make predictions about future events	• Electronic Medical Records and Genomics (eMERGE) network
	Genomic data are linked to phenotypic data already contained within clinical records	• IMPACT Model
		• CRASH model
Microbiome	The human microbiome produces metabolites that can modulate (on/off switch) gene expression	Acute brain injury modifies the immune system and also affects the composition of the microbiome, although the implications of these effects are not well established
	The intestinal microbiome regulates the lymphocyte population and plays a role in eliciting the immune response to acute brain injury	Fecal transplants for neuroprotection in TBI. (Benakis et al., 2016)
Neuromonitoring	Cerebral autoregulation-guided management	PRx assesses the dynamic component of autoregulation and is defined as the moving correlation coefficient between slow waves in intracranial pressure and arterial blood pressure. (Available Software: ICM+, Cambridge Enterprise, University of Cambridge, United Kingdom)
	ICP-guided management	Standard threshold for initiation of therapy is ICP ≥ 22 mm Hg
		The management of ICP should be based on individual injury patterns, ICP values and waveform analysis, brain compliance, clinical status, and head CT findings
		The Collaborative European Neuro Trauma Effectiveness Research in Traumatic Brain Injury (CENTER-TBI) group evaluated the ability to derive individualized ICP epidemiological thresholds and identify the impact of the “dose” above the individual ICP threshold on global patient outcomes
		ICP waveform analysis can provide insights into the compliance and elastance of the injured brain
	Multimodal neuromonitoring	Allows the understanding of concurrent global changes in the brain and physiological derangements in an individualized manner
	• Integration of information from the simultaneous monitoring of multiple physiological variables, including ICP, CPP, PRx, cerebral oxygenation, and microdialysis	
Other determinants	Sex	Female sex is associated with a higher incidence of a SAH and higher risk of DCI and mortality
	Sex hormones such as estrogen and progesterone affect neuronal pathways and modulate the immune system	
	Psychological traits	Baseline personality traits and psychological features are increasingly being recognized as important factors that affect psychosocial recovery and overall outcomes after acute brain injury. Hope, optimism, adaptive behavior, grit and resilience have been associated with improved psychosocial functioning after TBI (Rabinowitz and Arnett, 2018).

UCH-L1^™^, Ubiquitin carboxyl-terminal hydrolase L1; GFAP, glial fibrillary acidic protein; SAH, Subarachnoid hemorrhage; DCI, Delayed cerebral ischemia; ICP, Intracranial pressure; CPP, cerebral perfusion pressure; PRx, pressure reactivity index.

### Large Gaps in Our Knowledge of, and ability to Treat TBI

Two validated outcome calculators based on large cohorts have been proposed: the analysis of a sample of >10,000 TBI patients who were enrolled in the Corticosteroid Randomization after Significant Head Injury (CRASH) clinical trial conducted in multiple countries in 2004 (Perel et al., 2008), and the International Mission for Prognosis and Clinical Trials in Traumatic Brain Injury (IMPACT) prognosis calculator which included patients with moderate and severe TBI (GCS ≤ 12) from eight randomized controlled trials and three observational studies conducted between 1984 and 1997 (including the CRASH study cohort) (Steyerberg et al., 2008). These prognostic models used baseline characteristics, age, motor score on admission, and pupillary reactivity in addition to computed tomography (CT) of the head characteristics (Marshall CT score), hypotension, hypoxia, glucose and hemoglobin to be of predictive value. Within the IMPACT data, the best performance was seen for the three observational studies, with area under the curve (AUCs) of 0.66–0.84. When CT classification and traumatic subarachnoid hemorrhage (tSAH) were considered as well, the performance increased but only marginally. This model was found to be of predictive value at 6 months for mortality and unfavorable outcome (Egea-Guerrero et al., 2018). Nonetheless, predicting favorable outcome remains a challenge. The model was also found to be more accurate when used in TBI patients of high-income countries. Also, the Marshall CT score, IMPACT and CRASH models overestimated unfavorable neurological outcome in patients with severe head injury who underwent early decompressive craniectomy (Charry et al., 2017). Another pitfall was that the Injury Severity Score (ISS) was not included in the model as it was not available for all the patients. Nonetheless, other external validations showed good discrimination for both, the CRASH and IMPACT models, with AUCs ranging from 0.65 to 0.89, and good overall calibration (Roozenbeek et al., 2012; Han et al., 2014; Maeda et al., 2019). More recently, the IMPACT model was tested in a Spanish cohort of patients with moderate to severe TBI admitted to a level 1 trauma center. The IMPACT model validates prediction of 6-month outcomes in this population (Egea-Guerrero et al., 2018). Furthermore, the Collaborative European NeuroTrauma Effectiveness Research in Traumatic Brain Injury (CENTER-TBI) core study assessed the performance of the three IMPACT and two CRASH model variants. Both, the IMPACT and CRASH models adequately identified patients at high risk for mortality or unfavorable outcome (Dijkland et al., 2021). When it comes to mild TBI (mTBI) or post concussive syndrome, there are currently no validated long-term prognostic models (Silverberg et al., 2017) also, there is no general consensus upon which cognitive endpoint must be used to assess victims of mTBI (Oldenburg et al., 2016; Silverberg et al., 2017).

Despite very promising preclinical data, RCTs of neuroprotective strategies in moderate and severe TBI have failed to demonstrate significant treatment effects. Several pharmacological neuroprotective agents have recently been studied with varying degrees of success. Agents such as progesterone and cyclosporine had shown promise in earlier phase II studies. The results of progesterone phase III trials have been published, and several new pharmacological agents have also been studied in humans with acute TBI. Cyclosporine phase III trials have not yet started. Pharmacological therapies tested in at least two studies include progesterone, ethanol, growth hormone (GH), erythropoietin, barbiturates, and statins showing mixed results. There is presently no pharmacological agent that will unequivocally improve clinical outcomes after TBI, while several agents have demonstrated promising clinical benefits for specific TBI patients the evidence to support widespread use remains weak.

The National Institute for Health and Clinical Excellence (NICE) produced the first version of its head injury guidelines in 2003. The recommendations were updated in 2007 to reflect new evidence. Amendments were made to the section about the transfer of patients to neurosurgical services (Barratt et al., 2010). Despite technical progress, such as wider availability of CT scanning and advances in specialized neurocritical care there has been little improvement in outcomes following TBI since 1994, including developed countries (Wallis and Guly, 2003).

The Canadian CT Head Rule (CCHR) (Stiell et al., 2001) and New Orleans Criteria (NOC) (Haydel et al., 2000) are previously developed clinical decision rules to guide CT use for patients with minor head injury and with Glasgow Coma Scale (GCS) scores of 13–15 for the CCHR and a GCS score of 15 for the NOC. However, uncertainty about the clinical performance of these “rules” exists. Both, CCHR and NOC have equivalent high sensitivities for need for neurosurgical intervention and clinically important brain injury, but the CCHR has higher specificity for important clinical outcomes than does the NOC, and its use may result in less CT scans performed. (Stiell et al., 2005; Bouida et al., 2013). Since the inception of these guidelines little has been added to guide the management and prognosticate clinical outcome in patient with mTBI.

Currently, the diagnosis of TBI in the acute setting is centered on neurological examination and neuroimaging tools such as CT scan and magnetic resonance imaging (MRI). Nonetheless, CT has low sensitivity to diffuse brain damage. In the other hand, MRI provides more information on the extent of diffuse injuries, but its use is largely restricted by cost and its limited availability. Better predictive models are needed, as the existing validated ones are more useful in prognosticating poor outcome (i.e., mortality) and do not include biomarkers, genomics, proteonomics, metabolomics, etc. Definitive diagnostic blood tests for TBI to guide diagnosis and treatment are currently extremely limited. In response to these challenges, there has been a growing interest in the use of biomarkers for TBI prognostication and classification.

S100B is a protein primarily expressed by astrocytes and was the first biomarker proposed for clinical use by the Scandinavian Neurotrauma Committee (Calcagnile et al., 2012). Another biomarker of astrocyte reactivity is GFAP, which is an intermediate filament protein that has been shown to distinguish patients with TBI with intracranial findings on head CT from those without with high accuracy. UCH-L1 is a protein abundantly found in neurons.

In February 2018, the US Food and Drug Administration approved the use of the brain trauma indicator (BTI), which is a ubiquitin-C-terminal-hydrolase-L1 (UCH-L1) and glial fibrillary acidic protein (GFAP) assay for determination of the clinical need of a CT scan after mild TBI (Administration, 2018). The test predicted which patients with mTBI had intracranial lesions with 97.5 percent accuracy, and patients without lesions 99.6 percent of the time (Bazarian et al., 2018). More recently, the FDA granted clearance for the i-STAT Alinity TBI plasma test which was developed by the US Department of Defense (DoD). The test simultaneously measures GFAP and UCH-L1 with a 95.8% sensitivity and >99% negative predictive value (Bazarian et al., 2021).

### The Goal of Precision Medicine in TBI

Our efforts in precision medicine must be directed to close the gaps in the existing predictive models through a better understanding of the biological and behavioral characteristics rendered by each individual patient after sustaining a head injury. TBI is frequently classified into mild, moderate, and severe based on the Glasgow Coma Scale (GCS) score (Teasdale and Jennett, 1974), it should be however represented as a continuum of injury, as the term “mild TBI” is misleading especially considering the victims of head injury initially categorized with GCS of 13–15, are at risk for psychological, cognitive and physical impairment as well as being at risk for diffuse axonal injury (Rimel et al., 1981; Barth et al., 1983; Alexander, 1995; Macciocchi et al., 1996; Millis et al., 2001; Stein et al., 2009). The field of sports and military personnel concussion biomarkers has amounted a body of research that supports the use of biomarkers in the diagnosis of TBI in both children and adults. A systematic review of clinical studies exploring biomarkers of brain injury following concussions related to sports revealed that 11 distinct biomarkers have been examined in over a dozen studies and S100β was the most frequently measured, followed by glial fibrillary acidic protein (GFAP), neuron-specific enolase (NSE), tau, neurofilament light protein (NFL), and amyloid protein. Brain-derived neurotrophic factor, creatinine kinase, and heart-type fatty acid binding protein have also been studied (Papa et al., 2015). A recent ambitious blood-based biomarker panel to risk-stratify mild TBI screened 87 serum biomarkers. Two models resulted. In the broad inclusive model, 72 kDa type IV collagenase, C-reactive protein, creatine kinase B type, fatty acid binding protein—heart, granulocyte-macrophage colony-stimulating factor and malondialdehyde modified low density lipoprotein significantly predicted injury visualized on CT. Questioning the predictive value of more “traditional” biomarkers of brain injury previously described (i.e. S100β) however, the study did not address long-term outcome (Sharma et al., 2017).

A timely and individualized management of patients following head trauma with biomarkers could provide a better chance to prevent further secondary or delayed injury. Biomarkers could also provide a useful point-of-care (POC) tool to screen victims at risk for clinical deterioration on the field and in the Emergency Center. Similarly, biomarkers may confer a more accurate prognostic value to existing predictive models for long-term clinical outcome. Lastly, biomarkers could assist in monitoring spontaneous recovery from injury or in monitoring response to future therapies. The ideal biomarker should be easily obtained with minimum discomfort or risk to the patient (i.e., urine or blood sample), with early return of reliable results for early commencement of therapy of management, and monitoring effectiveness is highly desirable. Ultimately, a dependable biomarker will have a detection method that is sensitive and specific and is highly reproducible among clinical laboratories or POC monitors. A handful of biomarkers have shown a correlation with number of hits to the head, acceleration/deceleration forces, post concussive symptoms, trauma to the body versus the head, and dynamics of different sports. While there are currently no validated biomarkers for mTBI or concussion, there is potential for biomarkers to provide diagnostic, prognostic, and monitoring information postinjury. They could also be combined with neuroimaging to assess injury evolution and recovery. Though there are several interesting potential candidate biomarkers for determining severity of concussion, validation of these markers is lacking. Our efforts should be directed in validating the utility and applicability of these biomarkers already studied.

### Precision Medicine Approach

Predictive modeling uses many analytical techniques such as data mining, statistics and probability, modeling, advanced machine learning, and artificial intelligence to make predictions about future events. To this point, the prognostic models described and validated in TBI are resulted from analyzing cohorts and their predefined variables (age, GCS, pupillary reactivity, etc.) only. There is a major opportunity to develop predictive modeling from databases that will stay continuously updated and allow for analysis of variables that are less used, or not used at all (prior brain scans, prior hospitalization outcomes or laboratory results, medication interactions, social and pediatric history, etc.). For instance, in 2013 Memorial Sloan-Kettering, IBM and WellPoint announced a partnership to use Watson software, the question answering computer system capable of answering questions posed in natural language. This event marked the first commercial application of artificial intelligence for utilization management decision in lung cancer treatment. The combination of new technologies (software and hardware) and the adoption of Electronic Medical records (EMR) accelerated by the government’s Meaningful Use Act is now potentially bringing us closer to building a more personalized predictive model in TBI.

Precision medicine must be data-driven, quantitative and it will require iterative computational approaches. It ideally will encompass the entirety of the BIOME, from the molecular (genome, epigenome, transcriptome, proteome, metabolome, microbiome) to detailed quantitative neuroimaging or brain mapping and quantitative phenotype assessments, as well as extraction/analysis of detailed EHR/EMR datasets. Potential applications of these data include risk stratification or early prediction, biomarker discovery, population identification for clinical trial enrolment, and maximizing resource use. On the other hand, increasing adoption of EHRs implies that, a considerable amount of data accessible for such purposes will be from a time period during which both the practices and the clinical use of EHRs are constantly evolving due to transformations in technology and incentives. This problem has been addressed acknowledging the implications of a phenomenon called “non-stationarity,” on predictive modeling. It has been demonstrated that “Non-stationarity” can lead to quite different conclusions regarding the relative merits of different models with respect to predictive power and calibration of their posterior probabilities (Jung and Shah, 2015).

### Electronic Health Record Phenotyping

Health care systems and therapies are perpetually in a state of flux. The method of plotting raw EHR data into eloquent medical concepts, or the job of learning the medically relevant characteristics of the data is referred to as EHR-based phenotyping (Hripcsak and Albers, 2013) Examples of such large-scale phenotyping efforts are illustrated by the Electronic Medical Records and Genomics (eMERGE) Network (McCarty et al., 2011). Nonetheless, the present state of high-throughput phenotyping cannot produce sizable amounts of candidate phenotypes that also reach adequate performance without human interpreted samples. There are currently several limitations for EHR-phenotyping namely, lack of standardization and interchangeability across institutions, and it still requires human intervention. *Limestone*, a nonnegative tensor factorization method to generate phenotype candidates without expert supervision has been proposed. *Limestone* has addressed the challenge of automation in EHR-phenotyping to obtain better predictive accuracy of patients at risk of heart failure (Ho et al., 2014). In addition, Predictive Analytics (PA), a *learning model* of advanced analytics*,* has been proposed as a way to build better predictive models. PA may integrate not only technology and statistical methods to search through massive amounts of information in the EHR to predict outcomes for individual patients, but it can also include the latest medical research published in peer-reviewed journals and databases to make predictions. The two major forms in which PA differs from traditional evidence-based medicine are: *1*) predictions are made for individuals and not for groups, *2*) it does not rely upon a normal (bell-shaped) distribution curve. There is skepticism in the use of PA to predict outcomes, and that is that individuals and biological process are influenced by their environment in uncountable forms, including the human factor (i.e., choices from health care providers, patients and families). The environment changes constantly and quickly, and the amount of variability is difficult to objectively measure. Similarly, measuring the impact of those changes is even more challenging. Another issue with relying in “big data” is that for instance, observational studies, paradoxically, have suggested that patients who received more aggressive treatment (i.e., endotracheal intubation) have greater morbidity and mortality. Predictive models may discount the fact that it is the severity of sickness and not the therapy implemented what accounts for the outcome. This may lead to a self-fulfilling prophecy by implementing a given prognostic model generated by “big-data” (Andersen et al., 2017; Angus, 2017).

### Concept of “Endotypes” or “Endophenotypes”

Endophenotype is an epidemiological term used to connect behavioral symptoms with more well-understood structural phenotypes associated with known genetic causes or with abnormal genetic testing. This concept is being increasingly used in developmental disabilities, particularly looking at highly heritable polygenetic conditions such as ADHD, autism, and many psychiatric disorders. To be considered an endophenotype, a biomarker must fulfill four criteria: *1*) it is associated with illness in the population; *2*) it is heritable; *3*) it is largely state independent (manifests in the individual whether or not the illness is active); and *4*) within families, endophenotype and illness cosegregate (Lee Gregory et al., 2015). TBI is one the most common human afflictions, contributing to long-term disability. Nascent data indicate that functional improvement or worsening can occur years after TBI, now being considered a risk factor for neurogenerative disorders. As mentioned before, TBI is a heterogeneous disease, in which a variety of injury subtypes and molecular mechanisms tend to overlap. In order to develop precision medicine approaches in which specific pathobiological mechanisms are targeted by adequate therapies, techniques to identify and measure these many subtypes are needed.

Brain mapping that defines injury *signatures* such as the extent, cell type(s) and location of the structures injured as a marker of TBI may help closing the gap faced by existing predictive models. Both, in the acute phase as well as longitudinally. For instance, axonal injury has long been identified as a predictor of outcome after brain injury including TBI, (traumatic axonal injury, TAI (Medana and Esiri, 2003). However, the burden and distribution of TAI is rarely integrated in prognostic models mainly due to cost and availability. It is known that lesions resulting from TAI tend to be disseminated in multiple areas within the central nervous system and those may also evolve dynamically over time (Sharp et al., 2014). Also, current thinking holds that white matter (WM) is uniquely vulnerable to TAI. However, clinically diagnosed mTBI is not always associated with WM DAI suggesting an undetected cortical process. Parvalbumin interneuron has been linked to neocortical network dysfunction in experimental TBI models (Vascak et al., 2017). These types of disruptions are prone to affect domains of function which are not limited to a particular anatomical location in the brain but are related to the connectivity of neural systems, (i.e., consciousness, attention, memory, etc.) known to be exquisitely vulnerable in individuals after TBI. The delineation of TAI has been altered by the introduction of novel MRI sequences such as diffusion-weighted imaging (DWI), diffusion tensor imaging (DTI), and susceptibility-weighted imaging (SWI). Also, the mapping of functional activation using functional MRI (fMRI), positron emission tomography (PET), electroencephalography (EEG) and magnetoencephalography (MEG) indicates alterations in connectivity which may help differentiate “cognitive phenotypes” (“endotypes” or “endophenotypes”) and classify outcome probabilities following TBI (Castellanos et al., 2010; Pandit et al., 2013).

Traumatic microvascular injury is a common but relatively understudied TBI endophenotype. Under normal conditions, the brain is dependent on a steady blood supply, which is accomplished by a large network of vessels across the cerebral tissue. Different cell types are distributed along this network that work together to regulate cerebral blood flow, vascular permeability, and micronutrient supply. Collectively, these vascular structures are termed the neurovascular unit (Shlosberg et al., 2010), which consists of the endothelial lining of blood vessels, pericytes and perivascular astrocytes. Capillary endothelium forms a monolayer consisting of adherens and tight junctions to form the blood-brain barrier, which forms a highly regulated barrier between the systemic vasculature and the brain parenchyma. Under normal conditions, all components of this unit work in synchrony to form an integrated system capable to respond to a constantly changing cerebral and systemic environment. This process, termed neurovascular coupling, warranties consistent blood flow and micronutrient supply across the BBB as a function of neuronal activity. Following brain injury, these normal patterns of communication among the elements of the neurovascular unit can be severely affected, leading to inappropriate changes in cerebral blood flow in response to metabolic demands from the injured brain. Defining these subtypes of TBI that share a common biological process but is not immediately observable externally may assist our ability to diagnose, treat and prognosticate TBI (Hannawi and Stevens, 2016). Describing the organizational structure and evolution of brain networks in space and time after brain injury using graph theory has also been proposed (Hart et al., 2016).

### Prognostic Enrichment and Predictive Enrichment Strategies

Enrichment strategies are central for optimizing clinical trials and implementing precision medicine. Enrichment uses patient characteristics to select a study population in which a therapy or intervention is more likely to be detected than in a random population. Prognostic enrichment strategies select patients with a greater likelihood of having a disease-related event. Whereas predictive enrichment strategies select patients who are more likely to respond to an intervention or drug based on a biological or physiologic mechanism. Enrichment strategies are especially applicable when selecting patients for treatment trials in highly heterogeneous syndromes, as in TBI. Evidently, in brain injury, an effective pharmacologic neuroprotective therapy continues to escape us. Given the limited class I evidence available, up until now clinical protocols and guidelines have been largely based on expert opinion, for monitoring (Le Roux et al., 2014a) and treatment (Carney et al., 2007; Carney et al., 2016) by the Brain Trauma Foundation, which guidelines where last revised in 2016. Data-driven discovery in TBI has potential to yield significant information from large, heterogeneous data sets to enhance potential for precision medicine. Bioinformatics advances in precision medicine are gaining momentum as biomedical researchers start to cope with tremendous aggregates of data generated by all areas of science in the era of “big-data.” Informatics tools are being developed in preclinical and clinical brain injury studies. However, there remains a shortage of user-friendly integration that can be applied to primary research data from complex brain disorders. Randomized controlled trials have not led to any identifiable major advances. Natural subgroups of patients can be identified using topological data analysis (TDA) for discovery in preclinical spinal cord injury and TBI predicted by the presence of specific genetic polymorphisms (Nielson et al., 2015; Nielson et al., 2017). Also, Head injury Serum markers and Multi-modalities for Assessing Response to Trauma (HeadSMART) was a 6-month prospective cohort study that aimed to examine the utility of blood-based biomarkers to aid in TBI diagnosis, while also collecting longitudinal data on cognitive and other neuropsychiatric symptoms to analyze the prognostic utility of these blood-based biomarkers (Peters et al., 2017). HeadSMART II is an ongoing study which proposes to collect data using a multi-modality approach including blood biomarkers, clinical ssessments, neurocognitive performance, and neuropsychological characteristics, to identify subjects with a mild TBI and their likelihood of chronic symptoms, with an estimated completion date of December 2021. The Collaborative European NeuroTrauma Effectiveness Research in TBI (CENTER-TBI) represents a focused European effort to advance the care of TBI patients. It is part of a larger global initiative InTBIR: International Initiative for Traumatic Brain Injury Research with ongoing projects in the United States, Europe and Canada. This initiative expects a deep impact in terms of treatment strategies including precision medicine and personalized management, health care policy, economy and improved health (Maas et al., 2015). Since the completion of patient recruitment in 2017, close to 200 “CENTER-TBI” related projects have been published. Of relevance are collaborations between CENTER-TBI and TRACK-TBI to externally validated imaging features in the management of mild TBI patients (Yuh et al., 2021), predictive models of intracranial hypertension (Carra et al., 2021), and more recently the effect of brain temperature in intracranial hypertension and cerebral perfusion pressure (Birg et al., 2021). Moreover, a recent well-designed study in U.S. Army soldiers with deployment-acquired TBI, failed to demonstrate any utility of a cross-phenotype high-resolution polygenic risk score (PRS) analysis of persistent post-concussive symptoms (PCS) to predict neurodegenerative and psychiatric disorders, suggesting that persistent PCS does not share genetic components with these traits (Polimanti et al., 2017).

### Data-Driven Methods vs Model-Based Methods

Fault detection, isolation, and recovery (FDIR) is a subfield of control engineering which concerns itself with monitoring a system, identifying when a fault has occurred, and pinpointing the type of fault and its location. Precision Medicine in TBI may borrow these techniques to improve recognition of patients at risk of clinical deterioration in the acute phase, long-term prognosis, need for subsequent testing or therapy, etc. that are typically prone to human error. A model-based residual generation scheme as well as a data-driven linear discriminant analysis approach are developed to solve the fault detection and isolation (FDI) problem even when faults occur in the presence of system uncertainty, disturbance and noise, such as in TBI (Gradisek et al., 2012).

### Advanced Clinical Trial Design

In 2010 during the National Neurotrauma Symposium, the Directorate-General for Research and Innovation of the European Commission and the National Institutes of Health/National Institute of Neurological Disorders and Stroke organized a workshop on comparative effectiveness research (CER) in TBI. While several single-center studies have reported benefits of a range of interventions (i.e., mannitol, hypothermia, and decompressive craniectomy), none of these results remained generalizable in multicenter RCTs. Moreover, substantial selection bias may have existed in reporting benefits in single-center studies. This has led to a decline in the number of clinical trials initiated in TBI in the last decade, particularly in neuroprotection. Some of the arguments to explain possible causes for translational failures in clinical neuroprotection in TBI includes: *1*) to implement more intermediate studies in larger gyrencephalic mammals instead of rodents only, *2*) the response to injury may in part be genetically determined, and much research will be needed in the areas of genomics and metabolomics to elucidate the wide variability in the response to head trauma. Premorbid (i.e., medications and diseases, etc.) *3*) prehospital stratification (i.e., hypotension, hypoxia, etc.) could also help in identifying individuals likely to benefit from certain interventions, and *4*) mechanistic targeting *via* monitoring with brain tissue oximetry and microdialysis may allow to distinguish different pathophysiological processes such as ischemia, type of edema, cerebral hypoxia, or mitochondrial dysfunction as each may respond to different interventions (Maas et al., 2012).

More importantly, traditional clinical trials that rely on a hypothesis-driven, model-based approach may be reductionist and not be suitable vehicles for providing answers to all the questions that we have. TBI is a complex disorder, therefore there is likely no particular element that is fully responsible for the disease outcome. In contrast, a systems biology approach aims to identify multiple factors that contribute to the disease. Systems biology approaches the intricate interfaces of these manifold variables in a multivariate, multidimensional fashion, over time. There is a call for the implementation of CER. CER aims to quantify variations in outcome and to relate these to the type of care and its constituent components in ordinary settings and broader populations (i.e., Center A vs Center B). The research question is whether the difference in outcome is due to disparities in the initial severity of the injury or to a difference in management efficacy (Timmons and Toms, 2012). A recent example of CER comes from the IMPACT study where the outcome after TBI differed substantially between centers (Maas et al., 2012).

## New Approaches to Increase Precision in TBI Detection, Diagnosis, Severity Assessment

### Novel, More Specific, Phenotype assessments (Eye Tracking, Virtual Reality, Tasks)

There has been an interest particularly in mTBI to develop novel reproducible screening tools to detect concussive syndromes. Precise voluntary conjugate eye movement is controlled simultaneously by multiple cortical and subcortical structures, and it relies in the integrity of their connections. TBI has long been associated with impaired disconjugate eye movement. It has only been recently quantitatively measured *via* eye tracking devices (Pearson et al., 2007; Samadani et al., 2015a; Samadani et al., 2015b). In the future, our goal must be directed towards standardizing the results from these devices and building and interface that is user-friendly so that it can easily be applied in the field (i.e., by EMS personnel, coaches, bystanders, etc.). These devices may potentially be used to monitor recovery. They may also help to predict the head injury “endotype” and severity based on the pattern of eye movement impairment. A completed but yet to be published study using eye-tracking technology (NCT02634944) tested a device in combat and non-combat environments.

Computerized neuropsychological test batteries currently available are used to monitor recovery in studies. However, they all measure different cognitive domains and can be lengthy, requiring some level of expertise to apply and interpret. One possible alternative, the King-Devick test has been proposed. The K-D test measures the speed of rapid number naming, it requires less than 2 min to administer, post-fight scores were used to assess for concussion in boxers and martial arts fighters (Galetta et al., 2011). Currently, the Immediate Post-Concussion Assessment and Cognitive Testing (ImPACT) and ImPACT Pediatric are the only FDA-approved medical tools that are intended to assess cognitive function following a possible concussion. There are no studies that include a combined used of handheld eye-tracking devices and abbreviated cognitive tasks to assess for concussion in the field. Also, whether individuals can “learn” to score higher on these tests upon repeated exposure and masking the severity of the injury remains unclear. There is a modest level of evidence to support the use of virtual reality-based rehabilitation therapy for traumatic brain injury to improve static and dynamic balance over time (Cuthbert et al., 2014).

### Brain Mapping With MRI

It has been estimated that up to 10% of published studies in mTBI have a human neuroimaging component, using virtually every available medical imaging modality. Those studies assess either functional and structural changes and its link to neuropsychological evaluations after head trauma. To date, no neuroimaging modality or pattern finding can be used in isolation to diagnose, treat or prognosticate TBI. A recent meta-analysis showed that there are consistent neuroimaging markers of structure and function using fMRI and DTI suggesting that frontal areas are more vulnerable to injury (Eierud et al., 2014). DTI is the most promising technique to detect the subtle changes that occur in mTBI by demonstrated by white matter anisotropy. DTI has also been used to monitor changes over time after mTBI (Niogi and Mukherjee, 2010; Mayer et al., 2011). Some of the challenges in neuroimaging in TBI are the wide variability in the neuropsychological testing used as well as the time-post injury for obtaining the imaging studies.

The brain experiences various electrophysiologic changes after TBI (i.e., diffuse slowing, hippocampal excitability, decreased seizure threshold, etc.). EEG has been used to detect changes in the acute injury, monitor evolution and recovery after TBI. The data obtained with qualitative EEG studies does not allow quantification of the wave frequency spectrum present in the brain. Implementation of Fourier Transform (FT) EEG quantifies frequency bands present in the brain, commonly referred to as Quantitative EEG (QEEG). Because many of the EEG changes associated with mTBI are subtle, QEEG may provide useful adjunct information about cerebral electrophysiology following mTBI. Much work has been done in describing the qEEG changes of mTBI however, questions have been raised concerning the strength of the results as well as possible financial conflicts of interest limiting its widespread use in mTBI. Future work most attempt to correlate the qEE findings to the neurobiology of mTBI with histopathology and neurimaging (Haneef et al., 2013). There is also been an interest in using qEEG biofeedback in TBI rehabilitation (Thornton and Carmody, 2009), but robust independent studies are needed.

Magnetoencephalography (MEG) has been commercially available for over a decade however, its clinical accessibility remains restricted, with only a few dozen centers using this technology. EEG and MEG signals originate from the same neurophysiological processes however, there are important differences. Magnetic fields are less distorted than electric fields by the skull and scalp, which results in a better spatial resolution of the MEG. MEG is more sensitive to superficial cortical activity, and it can also be localized with more accuracy. There have been efforts to diagnose mild and moderate TBI using MEG (Huang et al., 2012; Tarapore et al., 2013; Lee and Huang, 2014) and in combination with MRI (DTI) (Huang et al., 2009). However, the evidence is limited and will likely remain so due to its limited availability.

### Real Time Multisignal Multisource Physiologic Data Capture Integration and analysis

The visualization dashboards of most existing monitoring devices cannot effectively present all physiological information of TBI patients. Most used monitor dashboards display only hemodynamic information. But they are not designed to facilitate a fast and accurate diagnosis by integrating neurophysiologic data (PRx, PbtO_2_, EEG, Optimal MAP, etc.) and their trends, which could guide and optimize clinicians’ diagnosis and prognosis decisions (Sebastian et al., 2012). With the advancement of Precision Medicine, the need for developing such analytic displays will become imperative.

## Neuromonitoring to Personalize TBI Therapy

Over the past half-century, multiple technological advances in critical care have led to substantial improvements in patient care, including the development of neurointensive care units. In these highly specialized hospital units, in addition to the clinical examination and monitoring technology routinely used, single or multiple neuromonitors are employed in the care of the acutely injured brain. The combined integration and interpretation of “trends” in these neuromonitoring variables is commonly referred as multimodal monitoring (MMM). Different invasive and noninvasive neuromonitoring technologies are available for clinical use, with normal ranges and treatment thresholds mostly defined by observational studies and expert consensus guidelines on multimodality neuromonitoring (Le Roux et al., 2014b).

The current management of TBI is centered in the concept that prevention of secondary insults (e.g., cerebral hypoxia, intracranial hypertension, subclinical seizures, etc.) will improve patient outcome. Unfortunately, these insults may not be promptly detected by clinical examination alone, especially in patients in whom the clinical features of the disease are clouded by standard treatment strategies such as sedatives and neuromuscular blockade. MMM offers an invaluable opportunity to supplement the clinical assessment to allow for early detection of secondary injuries through simultaneous measurement of different cerebral physiologic variables such as cerebral blood flow and metabolism, oxygenation and autoregulation. Thus, treatment strategies can be tailored in-real time based on changes in neurophysiology rather than by predefined standardized thresholds.

Nonetheless, the level of evidence to support its widespread use remains insufficient. In large part because the studies conducted are observational. There continues to be debate in *1*) the ideal location for the probe, *2*) establishing physiological thresholds to implement therapy and, *3*) which therapy should be implemented. Similarly, there is a lack of clinical outcomes. Future studies must be directed towards answering these questions. There is also dispute on the value of the data obtained and whether it is representative enough of the pathophysiological state of the injured brain. Existing brain tissue oxygenation and microdialysis probes sample only a very small volume of tissue around the probe. The readings can easily be affected by the location of the probe (i.e. “normal” appearing brain vs contusion vs penumbra) (Ponce et al., 2012). Some argue that determination of the utility of PbO_2_ monitoring can only be accomplished by blinding bedside clinicians to the results of this test, this hypothesis was tested in the Brain Tissue Oxygen Monitoring in Traumatic Brain Injury clinical trial (BOOST 2; clinicaltrials.gov identifier NCT00974259). BOOST-2 demonstrated that patients monitored with a combination of P_bt_O_2_ and ICP, had shorter periods of cerebral hypoxemia (P_bt_O_2_ <20 mmHg) compared to those monitored with ICP catheters only (Okonkwo et al., 2017).

### Intracranial Pressure Monitoring

The utility of invasive ICP monitoring to assist in the management of severe TBI patients was questioned by the results of the BEST TRIP trial. The authors concluded that in patients enrolled in the study, management focused on maintaining monitored intracranial pressure at 20 mm Hg or less was not shown to be superior to care based on imaging and clinical examination (Chesnut et al., 2012). Its publication has resulted in substantial controversy in the treatment of severe TBI. The Brain Trauma Foundation guidelines for the management of severe TBI patients hold as a level II recommendation that ICP should be monitored in “*all salvageable patients with a severe TBI and an abnormal CT scan*.” The best trip trial was not designed to measure the efficacy of ICP monitoring but to compare two management protocols for treatment of severe TBI: one involving ICP monitoring and the other involving serial CT imaging and neurologic examination. Further investigation is necessary in the areas of selection of patients for ICP monitoring, determination of patient specific ICP thresholds, and development of treatment methods and paradigms (Chesnut et al., 2015). While the trial demonstrated the feasibility of clinical management in low-socioeconomic areas, the results are not considered to be generalizable in other settings. There are claims that this study has a low external validity, due to concerns regarding generalizability as well as ethical and methodological considerations (Sahuquillo and Biestro, 2014).

On the other hand, other registry-based studies have demonstrated decreased in-hospital mortality when ICP-guided therapy is employed (Farahvar et al., 2012; Agrawal et al., 2016). Despite evidence of potential mortality benefits from ICP guided therapy, it has been reported that less than 50% of patients eligible for ICP monitoring receive an ICP monitoring catheter (Dawes et al., 2015; MacLaughlin et al., 2015). Blind trust on absolute ICP thresholds as recommended by clinical guidelines ignore the heterogeneity of physiologic changes after TBI, as well as unique subject’s characteristics and responses. Thus, it is now recognized that treatment interventions can be better optimized by individualized interpretation of ICP values along with other neuromonitoring variables, injury characteristics, and weighing the potential benefits and risks of specific treatment modalities (Kirkman and Smith, 2014).

More recently, researchers have tried to “predict” ICP elevations using ICP values or a combination of physiologic data (ICP and mean arterial pressure) (Güiza et al., 2017). In a retrospective analysis of TBI patients, an automated computer algorithm was able to predict ICP crises with 30 min advance warning from previous ICP measurements and time since last episode of elevated ICP (Myers et al., 2016). Recognizing the importance of secondary insults prevention, the addition of this class of algorithms to the bedside armamentarium can aid the clinician to minimize these insults *via* the early recognition of episodes of intracranial hypertension.

### Cerebral Oxygenation

Brain tissue oxygenation may be affected by many variables such as CPP, hemoglobin concentration, oxygen saturation, metabolic rate and cerebral vasospasm. It provides information about the fine balance between cerebral oxygen delivery and consumption and ultimately the adequacy of cerebral perfusion (Kirkman and Smith, 2016). Brain tissue oxygen can be measured using multiple approaches such as oxygen-15 PET, brain tissue oxygen partial pressure (P_bt_O_2_), spectroscopy, jugular bulb venous oximetry (SjvO_2_) and near-infrared spectroscopy (Yang, 2020).

#### Jugular Venous Oxygen Saturation

SjvO_2_ can be measured by intermittent blood sampling from a catheter positioned in the jugular bulb or continuously using a fiberoptic catheter. Sjvo_2_ reflects the global balance between cerebral oxygen delivery (supply) and the cerebral metabolic rate of oxygen (demand). Normal SjvO2 values range from 55 to 75% (Senapathi et al., 2017) and jugular venous desaturation has been associated with worse outcomes post TBI (Smith, 2018). Current guidelines recommend maintaining SjvO2 >50% in order to improve outcomes in the TBI population (Carney et al., 2017), acknowledging the fact that SjvO2 >75% has also been associated with poor neurological outcomes (Senapathi et al., 2017; Smith, 2018). Sjvo_2_ provides only information of a global state of cerebral oxygenation, and focal ischemic areas are not evaluated with this technique. Restricted oxygen diffusion or extraction due to neuronal infarction or inflammation, decreased cerebral metabolism and hyperemia are among the etiologies for increased Sjvo_2_. On the other hand, local or systemic hypoperfusion (e.g., intracranial hypertension, shock or prolonged hypotension, vasospasm), decreased systemic oxygen supply (e.g., low PaO_2_), and increased cerebral metabolism or oxygen extraction (e.g., seizures, fever) can cause reductions in Sjvo_2_. Given its invasive nature, complex interpretation, and being technically challenging, SjvO_2_ clinical use has decreased in favor of other methods of monitoring brain tissue oxygenation (Stocchetti et al., 2015).

#### Brain Tissue Oxygen Partial Pressure (PbtO_2_)

The most commonly used intraparenchymal oxygen sensor is the Licox monitoring system (Integra NeuroScience, Plainsboro, NJ, United States) which measures regional brain tissue oxygen tension and temperature. This technique uses a catheter that is placed preferably in the penumbra of the injured brain (Ponce et al., 2012), although such precise positioning can sometimes result challenging or even impossible. This argues for its placement on normal viable tissue where it may reflect global oxygenation (Ponce et al., 2012). PbtO_2_ is a highly complex variable influenced by many factors such as ICP, CBF, inspired fraction of oxygen (FiO_2_), hemoglobin values, cerebral autoregulation and metabolism. Thus, normalization or worsening of any of those variables can affect PbtO_2_. It is recognized that a “normal” PbtO_2_ can be greater than 15–20 mmHg and lower values may indicate cerebral hypoxia and are associated with a greater lactate/glucose ratio and increased glycerol levels. However, there is no universal consensus on the threshold at with tissue injury occurs (Casault et al., 2020).

Many studies have demonstrated an association between low PbtO_2_ and worse outcomes after TBI (Spiotta et al., 2010; Lin et al., 2015). Randomized controlled trials directed to study whether P_bt_O_2_ and ICP monitoring together provide better clinical outcomes than ICP monitoring alone have shown promising results. Among these, Brain Oxygen Optimization in Severe TBI-(BOOST-2) trial demonstrated that patients in the P_bt_O_2_ and ICP monitoring group had shorter periods of cerebral hypoxemia (P_bt_O_2_ <20 mmHg) compared to those in the ICP only monitoring group (Okonkwo et al., 2017). BOOST-3 is the multicenter version of this trial, and patient enrollment is currently ongoing. Another prospective multicenter study of 50 patients with moderate and severe TBI, PbtO_2_/ICP–guided therapy was associated with a significant reduction in mortality at 3 and 6-month after injury compared to ICP–guided therapy alone (Lin et al., 2015).

Brain tissue hypoxia can be reversed *via* many interventions including blood pressure augmentation, FiO_2_ increments, blood transfusion and optimization of ICP and CPP. Although probably erroneous, incremental increases in FiO_2_ are likely the most popular intervention. However, reliance on this technique is unlikely to be a permanent solution as further and deeper pathophysiologic changes occur in the acutely injured brain. Moreover, hyperoxia can lead to cerebral excitotoxicity (Quintard et al., 2015). Also, administration of sedatives, anti-inflammatory medications and cooling techniques are used since agitation, shivering, and fever can increase brain metabolism and further affect oxygen delivery (Cecil et al., 2011).

Despite the growing evidence in favor of the use of PbtO_2_ directed therapy in TBI, it was not until recently that the Seattle International Severe Traumatic Brain Injury Consensus Conference (SIBICC) released a class-III tiered-algorithm for the management of TBI patients based on both ICP and P_bt_O_2_ monitoring. The treatment groups are divided into four based on their ICP and P_bt_O_2_ and different treatment suggestions according to the group in which they were categorized were provided: Type A (normal ICP and P_bt_O_2_), Type B (elevated ICP and normal P_bt_O_2_), Type C (normal ICP and low P_bt_O_2_) and Type D (elevated ICP and low P_bt_O_2_) (Chesnut et al., 2020).

#### Near-Infrared Spectroscopy

Cerebral near-infrared spectroscopy (NIRS) has been proposed as a viable, non-invasive method to assess for the presence of intracranial hematomas and an alternative to invasive brain oximetry monitoring procedures. NIRS systems are usually portable, handheld devices that measure the optical attenuation of the NIR wavelength. The non-invasive nature of the device and its portability make NIRS a considerable tool for the evaluation of the brain injured patient. Because living tissue will absorb light in the 600–1,000 nm wavelength band differently based on the level of oxygen saturation in blood, monitors based on measuring the attenuation of reflected light have long provided measurement of peripheral oxygen saturation. Spatial resolution is limited by the depth of light penetration and further reduced by factors such as skull thickness, spatial postural position, and cerebrospinal fluid. As with many new monitoring technologies, the most significant obstacle to clinical implementation is a lack of evidence that decisions based on the output of the device can improve patient outcomes. Limited evidence exists regarding use of NIRS-based technology when determining outcomes. The rSO_2_ values have been determined to be a stronger discriminator and a predictor of hospital mortality when compared against the traditional parameters such as admission GCS, glycemia or hemoglobin levels. In a prior study, when rSO_2_ values at 1 h after ICU admission did not exceed 68% in the left hemisphere and 68.3% in the right hemisphere, the hazard ratio for death increased by 17 times (*p* < 0.01) and 5.1 times (*p* < 0.05) respectively (Vilke et al., 2014). Another study found that NIRS-derived PRx showed good ability to correlate with outcomes in TBI (Zweifel et al., 2008). Other evidence has shown NIRS as a promising method of assessing cerebral autoregulation when used in concert with systemic blood pressure and ICP monitors (Rivera-Lara et al., 2017). Further research is warranted in this area as non-invasive neuromonitoring technology becomes more available and reliable.

### Cerebral Microdialysis (CMD)

Along with the other monitoring parameters currently available, several studies have revealed the importance of monitoring the metabolic state of the brain. This can be achieved using CMD which measures metabolite concentrations within the brain’s extracellular space, such as glucose, lactate, pyruvate and glutamate. This technique involves the invasive insertion of a probe with a 10-mm length semipermeable distal end membrane that is positioned 2 cm deep into the pericontusional penumbra within the brain parenchyma. This catheter is pumped with isotonic fluid that diffuses into the interstitial space and by a diffusion principle, metabolites are recovered and analyzed (Cecil et al., 2011).

**FIGURE 1:**
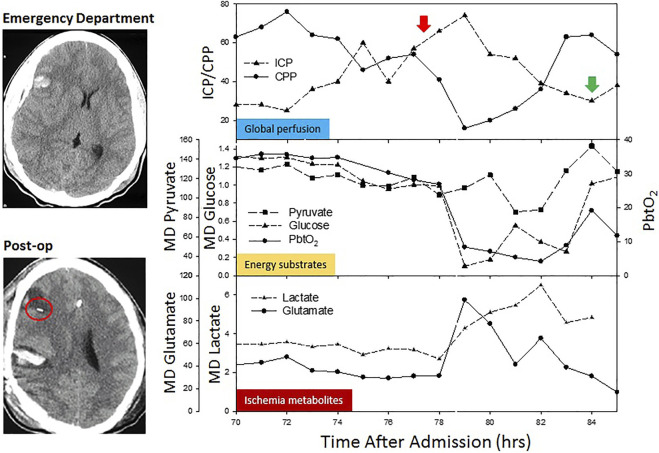
Classic neurophysiologic changes observed during an acute episode of increased intracranial pressure in a patient undergoing multimodal monitoring. A computed tomography demonstrates pre- and post-MMM probes placement in the penumbra of the intracerebral contusion. As ICP increases (red arrow) and CPP drops, there is a reduction in energy substrates (glucose and PbtO_2_), with a parallel elevation in ischemia metabolites (lactate and glutamate). As the ICP crisis is temporized (green arrow), energy substrates and ischemia metabolites return to pre-crisis values.

During states of hypoxia and ischemia after sTBI, the Krebs’ cycle end products become affected. Since their levels normally fluctuate, it is believed that a good indicator of the brain tissue metabolic state, energy failure and mitochondrial dysfunction is the lactate-to-pyruvate ratio (LPR). CMD aims to identify metabolite alterations so that interventions can be performed in real-time that will possibly allow normalization of abnormal patterns. Figure 1 demonstrates a typical pattern observed after acute brain injury including increased lactate and pyruvate and low glucose, associated with low PbtO2 suggesting further secondary damage and has been associated with increased mortality (Cecil et al., 2011).

Data from PET and CT perfusion studies have shown that ischemia does not happen as frequently as previously thought. In nonischemic scenarios, the metabolic disruption seems to be related to increased lactate secondary to increased glycolysis (Vespa et al., 2005). On the contrary, CMD technique has helped to describe ischemic metabolic crisis which involves low cerebral glucose (<0.7 mmol/L) related to decreased CBF (<35 ml/100 g/min), which ultimately leads to further injury (Sala et al., 2013; Bouzat et al., 2015).

Ultimately, the use of CMD focuses on understanding the pathological metabolic imbalances that arise after TBI and it seems promising to establish further courses of action and individualize therapy (Vespa et al., 2007; Stocchetti et al., 2013; Kurtz and Rocha, 2020). However, despite evidence from several studies confirming that abnormal brain chemistry relates to poor outcomes after TBI, the clinical utility of CMD-guided therapy remains a debate (Hutchinson et al., 2015).

### Cerebral Autoregulation

#### Non-invasive: Optimal MAP/CPP

Cerebral autoregulation is the ability of the cerebral vascular bed to maintain a steady flow despite the effect of arterial blood pressure (ABP) variability. This capability may be lost in TBI leaving the still viable brain tissue at risk against the effect of acute ABP changes. Precise autoregulation can be evaluated by analyzing any changes in cerebral blood flow (CBF), cerebral perfusion pressure (CPP) or mean arterial pressure (MAP). For instance, when autoregulation is compromised, hypotension reduces CBF and worsens ischemia. Contrarily, high ABP causes increases in ICP and CBF. CBF is dependent on CPP and is inversely proportional to cerebrovascular resistance. Normal CBF is 18–35 ml/100 g per minute and its critical threshold value is 15 ml/100 g per minute (Botteri et al., 2008).

Manipulation of blood pressure is a mainstay of therapy in patients with acute brain injury. Several studies in adults have shown the feasibility to individualize MAP and CPP goals by using cerebral autoregulation monitoring for calculating optimal CPP or optimal MAP. Some studies have shown that patients in whom median CPP or MAP differed significantly from optimal CPP or optimal MAP, defined by cerebral autoregulation monitoring, were more likely to have an unfavorable outcome. There are over a dozen Cerebral Autoregulation indices. Some measure autoregulation (COx, TOx, CFVx, Sx, Mx-ABP, Mx-CPP and ORx), while others measure cerebrovascular reactivity (PRx, HVx, THI and ARI). The pressure reactivity index (PRx) is probably the most known method to assess cerebral autoregulation. It is calculated as the moving Pearson correlation coefficient between 30 consecutive, 10-s averaged values of ICP and ABP over a 4-min period and ranges between −1 and +1 (Aries et al., 2012). An inverse correlation between ABP and ICP, indicated by a negative PRx value, represents normal cerebrovascular reactivity, whereas an increasingly positive PRx defines a continuum of increasingly nonreactive cerebrovascular responses. These changes in PRx can be calculated and then used to find the CPP value at which cerebral perfusion may be optimal. Targeting optimal CPP rather a generic CPP threshold probably reduces the risks of suboptimal CPP and has been associated with improved outcomes (Aries et al., 2012; Dias et al., 2015). Cerebral autoregulation can also be assessed using an oxygen reactivity index and ABP (Jaeger et al., 2006), the correlation between ABP and transcranial Doppler-derived mean blood flow velocity (Sorrentino et al., 2011) or wavelet analysis of slow wave oscillations in ABP and near infrared spectroscopy-derived cerebral hemodynamic variables (Highton et al., 2015).

Targeting optimal CPP seems a logic approach in TBI management, unfortunately there are no good-quality clinical trials to support its routine bedside application (Needham et al., 2017). Moreover, this technology presents many challenges as methods of calculating the PRx and other autoregulatory indices require expensive and time-consuming high-frequency signal processing and automated analysis.

Monitoring systems for CBF are still in progress and need further development, but some are currently available. Although transcranial Doppler sonography’s application has mainly being used in the care of patients suffering from subarachnoid hemorrhage, it has been used to provide a real-time assessment of changes in flow velocity and detection of posttraumatic complications such as hyperemia, vasospasm, low CBF and ICP variability in TBI (Saqqur et al., 2007; Cecil et al., 2011). The Hemedex CBF monitoring system (Codman & Shurtleff, Inc.) uses a principle of thermal conduction, in which tissue perfusion at a capillary level using a probe is determined by calculating thermal convection and total dissipated initial power. The probe is inserted in uninjured areas of cerebral parenchyma or in the injury’s “penumbra” (Raynham, 2008; Cecil et al., 2011).

Pressure reactivity index (PRx) is a commonly used index in research and in clinical practice. PRx is the correlation coefficient between ICP and arterial pressure therefore it requires invasive ICP monitoring. Other indices have been described in patients that did not require ICP monitoring during acute brain injury using near-infrared spectroscopy (NIRS) and/or ultrasound Doppler. The influence of CPP on autoregulation has led to finding that the CPP at which autoregulation is best preserved (CPP_opt_) varies both among individuals, and throughout time in an individual patient (Czosnyka et al., 2001). Leading to think that there is a potential for personalized management of blood pressured in acute TBI victims. A recent systematic review proposes that even though the data indicate an association between variation from CPPopt and poor clinical outcome at 6 months, the level of evidence precludes safe conclusions (Needham et al., 2017). Further research should be directed towards prospective, randomized controlled studies to clarify its role in the management of TBI in the acute phase. Also, much research is needed to help elucidating which of the many different indices is most useful when managing patients with severe TBI, invasively and non-invasively. And whether some of these indices are equivalent and reliably interchangeable when ICP monitoring is not indicated. Validation studies in humans are lacking.

### Multimodal Neuromonitoring Integration

MMM faces an enormous challenge because of the number of interactions and complexity of physiologic variables, real-time data analysis and artifact reduction. Thus, computational analysis and integration are essential to make MMM viable and accessible to any hospital ICU. Commercial systems that allow processing, analysis and data integration are currently available. The CNS monitor (Moberg Research Inc.) allows individualized medical care by integrating different physiologic parameters from different interfaces. By following trends in physiologic variables, the provider is allowed to intervene in real-time.

To date, innumerable studies have been published assessing the effect on outcome of individual neuromonitors after severe TBI. However, no trial has been performed to assess the effect of MMM as a whole in the management of TBI. The TBI-Multimodal Monitoring Study (monTBI) trial is an ongoing British trial comparing the outcome effect of MMM (ICP monitoring, cerebral metabolism, brain tissue oxygen concentration, biomarkers and cytokine concentration) after sTBI. This study may give us a deeper understanding of the utility of MMM in the management of TBI.

## Traumatic Brain Injury and Its Distinct Injury Patterns

A CT with positive results for acute intracranial hemorrhage is the gold-standard diagnostic biomarker in acute traumatic brain injury. Several studies have shown that positive head CT results in worse outcomes and TBI. Associations between individual CT scan findings and outcomes have been reported in mild, moderate, and severe TBI (Marshall et al., 1991; Maas et al., 2005; Marmarou et al., 2007). It is critical to understand that a positive head CT involves a wide spectrum of possible intracranial findings that can’t be categorized as a “one size fits all” approach. Moreover, TBI should not be categorized as a disease but rather as a syndrome encompassing many individual injury patterns. Therefore, a more precise understanding of CT abnormalities in TBI, beyond the simple presence or absence of intracranial lesions on CT is critical. It was not until recently that this concept was further studied. In a study derived from the TRACK-TBI cohort of mild TBI subjects, pathological CT features carried different prognostic implications after mild TBI to 1-year postinjury (Yuh et al., 2021). Some patterns of injury were associated with worse outcomes than others. In this study, while reconfirming the importance of patient baseline characteristics in mTBI outcome, authors demonstrated for the first time that different pathological subtypes of intracranial hemorrhage are not equivalent in their implications for prognosis. This finding of varying odds ratios for different subtypes of intracranial hemorrhage, including high odds ratios for intraventricular hemorrhage and petechial hemorrhage as markers for rotational injury, appears to be a new observation in mTBI that deserves further evaluation. Based on large observational studies, cerebral contusion, subarachnoid hemorrhage, subdural hematoma, intraventricular hemorrhage, and petechial hemorrhage are associated with adverse outcomes across a broad range of GOSE scores up to 1 year after mTBI, while epidural hematoma is not. These easily identifiable imaging findings can be used to stratify patients at risk for unfavorable outcomes and likely to improve clinical trial design. These findings support the idea that patients with TBI and specific CT features should be considered for guided education and systematic follow-up.

## Genomic, Epigenomics, and Transcriptomics in Traumatic Brain Injury Research

The heterogeneous nature of TBI with a multifaceted biological response, and substantial unpredictability in human rehabilitation contributes to the difficulty in identifying therapeutics that improve outcomes. In an era of Precision medicine, the progress in medical genetics and genomics over the past two decades have boosted our understanding of many biological processes and even outcome after TBI, though this research has been mainly preclinical.

### Genomics and Epigenomics

Though Genetics studies the changes in deoxyribonucleic acid (DNA) sequence for single-gene disorders and it may be useful in some multifactorial disorders, Genomics on the other hand is a method approach to the daunting task of studying gene variability across the entire genome of groups of individuals from a population of interest, comparing to a similar control group. Genomics studies the genome differences within groups and disease states that lead to variability in recovery in a complex biological system, such as TBI.

Modern systems biology use Genome-wide association studies (GWAS) to gain a thorough view of the impact of TBI on central aspects of gene regulation, which have the potential to determine or change the course of the TBI pathology. In an experimental model, it has been shown that TBI perturbs epigenomic programming, transcriptional activities, and the organization of genes in networks centered around genes such as Anax2, Ogn, and Fmod (Meng et al., 2017). The homology between “genomic signatures” (characteristic frequency of oligonucleotides in a genome sequence) from blood and brain elicited by TBI provides proof of concept information for development of biomarkers of TBI based on composite genomic arrays. This shows that TBI can reprogram genes which could result in predisposition to neurological and psychiatric disorders, and that genomic information from peripheral leukocytes can also predict TBI pathogenesis in the brain.

The GWAS method produces multiple points of potential genetic variability in people with the outcome variable in question. Currently there are commercially available methods for GWAS studies. Important decisions to be made at the design stage of these studies are, the choice of the commercial genotyping chip to be used and the numbers of case and control samples to be genotyped. Measure of coverage is the most common method to comparing different chips however, this method is flawed. Another more effective method, genotype imputation, has been proposed (Spencer et al., 2009). Leading to the argument that when taking budgetary considerations into account, the most powerful design may not always correspond to the chip with the highest and ideal coverage. Future work should be directed towards building a specific chip for the study of TBI. To be informative, GWAS studies with adequate design need also a phenotype that is well defined (Clarke et al., 2011). The groups compared have to be similar in all aspects except the outcome, proving especially challenging in TBI (Hicks et al., 2013). Numerous variables, such as gender, age, and type of injury, must be controlled for in the analysis, adding to the difficulty in the statistical method needed. The principle of studying gene-gene interactions using GWAS is that the effect of variation within two or more genes of interest is greater than the effect of either gene alone. *A priori* selection of genes to be studied may help saving cost and reducing the likelihood of false positives. For instance, one study examined the relation of SNPs in the glutamic acid decarboxylase (GAD) genes, GAD1 and GAD2, and the risk of seizures after TBI in humans (Darrah et al., 2013).

To date, there has been minimal research using genomics in TBI. It is fundamental to standardize phenotype data collection, use standard data elements, and create databases to allow for multicenter research studies combining samples to effectively power genomic research. The Federal Interagency Traumatic Brain Injury Research (FITBIR) informatics system database initiatives provide an answer to these efforts with a directive from the National Institutes of Health in TBI research to register data into this database for future cumulative analyses.

Epigenomics approach focuses on determining nonsequence variation (i.e., histone modification, methylation, and miRNA [micro-RNA]) modifying gene transcription, translation, and RNA stability of a given gene/gene product. These changes are different for different cell types and environments and are subject to modification by various factors such as maternal behavior, exercise, diet, aging, etc. Substantial causes of modification to epigenomic processes over the course of an individual life span makes epigenomics particularly challenging to study (Pearson and Manolio, 2008). The International Human Epigenome Consortium (IHEC) is a resource for investigators, providing a series of data sets of reference epigenomes. IHEC sets quality standards and offers efficient communication structures, promoting constant exchange among scientists.

### Transcriptomics and Proteonomics

At a given moment in time in a disease or health state and in different cell types, different genes are silenced or transcribed at different rates. The study of the total set of ribonucleic acid (RNA) transcripts from a given sample is called Transcriptomics. It focuses on the product of one gene’s transcription or the transcription products of multiple genes with a single or multiple pathways. Computational methods for analyzing gene expression leads to identifying differences and interpreting results (Merrick et al., 2011). Most studies examining gene expression in TBI have been conducted in experimental models. For instance, high mobility group box protein-1 (HMGB1), Galectin-3 and NOS2 have been implicated in cerebral edema, immune response, and microglia activation respectively, after TBI in animal models (Laird et al., 2014; Kurland et al., 2016; Yip et al., 2017). Another experimental model showed activation of the 2′, 3′ cAMP-adenosine pathway *via* brain microdialysis (Verrier et al., 2011) which was later translated to human research using CSF from repository samples from severe TBI victims (Verrier et al., 2012).

Evidently it is not always feasible to obtain brain tissue samples to study the pathophysiology of TBI. Peripheral leukocytes may offer a measurable correlate as demonstrated by down-regulation of olfactory receptors (OR11H1 and OR4M1) in blood cells following traumatic brain injury which also correlated with the severity of brain injury and TBI-specific symptoms (Zhao et al., 2013). It can be argued that such findings could: *1*) predict memory loss and tauopathy after TBI, *2*) help establishing severity of disease, and *3*) improve patient selection in TBI for clinical trials. Larger samples of patients after TBI are needed to corroborate these findings.

## Behavioral Biomarkers in Traumatic Brain Injury

TBI has traditionally been associated with cognitive and behavioral changes and neuropsychiatric sequelae during both the acute and chronic phases of injury. It has been well established that majority of psychiatric disorders without comorbid brain injury are closely associated with neurobiological changes to mesocortical and mesolimbic circuitry, receptor properties, and/or dysregulation of the hypothalamic-pituitary-adrenal axis. Thus, it is likely that preinjury psychiatric disorders prime the brain for a more severe neurometabolic postinjury cascade. As a result of these biomechanical factors, TBI preferentially affects the same core mesocortical and mesolimbic circuitry that has been implicated in seminal reviews of depression (Ferrari and Villa, 2017), post-traumatic stress disorder (PTSD) (Fenster et al., 2018), and cognitive functioning. In general, the severity, number and types of previous mild TBIs and their proximity (i.e., clustered together) and extracranial injuries represent the main injury factors influencing outcomes important injury factors. A history of concussion not only increases the risk of future concussions, but also increases baseline symptoms, as well as long-term cognitive and psychiatric dysregulation in athletes (Harmon et al., 2013).

The effects of repetitive mild TBI have been implicated in an increase in neurodegenerative disease (Lehman et al., 2012) and the accumulation of tau in perivascular spaces in deep cortical sulci (Bieniek et al., 2015). As such, a detailed history of previous organized contact or collision sports participation (i.e., exposure history) represents a critical part of the clinical evaluation of chronic symptomatology. It is vital to recognize that recovery after mild TBI does not represent a unitary concept as frequently conceptualized by most clinicians. Deficits on cognitive testing may differ when assessing somatic versus cognitive symptoms. Similarly, current imaging biomarker data suggest a complex, differential pattern of resolution occurring over weeks or months, depending on the biomarker (Meier et al., 2015). Therefore, defining “recovery” based on single variables carries the risk to premature clinical decisions that can place patients at risk, especially in sport related injuries where the risk of repeated trauma is latent. Although the majority of single-episode, uncomplicated mild TBI resolve within days to weeks, most of traditional neurobehavioral measures 3–6 months after injury are unable to detect impairment in up to 95% of subjects (Karr et al., 2014). Thus, as our ability to promptly detect and diagnose deficits, our understanding of the true “recovery”after TBI will continue to evolve.

Factor associated with prolonged post-concussive syndrome (PCS) have been identified including psychiatric illness, low degree of education, learning disability at baseline, and very young or elderly status (Ponsford et al., 2011). Depression represents the most commonly diagnosed psychiatric disorder after TBI in its varying degrees, whereas PTSD is highly comorbid in military personnel. Substance use and abuse increase the risk of sustaining a TBI, with up to 50% of mild TBI occurring under the influence of alcohol (Scheenen et al., 2016). Psychiatric illness preceding TBI, including family history of mood disorders, and history of substance use, are strong predictors of presence/severity of PCS. Affective dysregulation or substance abuse following mild TBI may result from changes in lifestyle indirectly associated with injury (Dikmen et al., 2004). Alternatively, mesocorticolimbic networks or their white matter connections may be affected by mild TBI (Bigler and Maxwell, 2012), leading to organically induced neuropsychiatric syndromes, which otherwise appear to be “purely” psychological in nature.

Patients unable to manage stressful situations are more likely to adopt maladaptive coping strategies after mild TBI, and usually experience worse PCS, including somatic symptom disorder and conversion disorder (Broshek et al., 2015). In summary, the importance of comprehensive phenotyping of postconcussion injury status, premorbid personal and family history, neuropsychological testing, advanced imaging, and laboratory examinations must be considered by clinicians in order to adopt a diagnostic framework to comprehensively approach the complex spectrum of TBI.

## Current Big Science Clinical and Translational Programs Dedicated to the Study of TBI

These rich and diverse precision medicine datasets reside in publicly accessible infrastructure of databases, imaging and biomarker repositories. The main goal of these international resources is to identify new diagnostic, therapeutic and prognostic markers and to refine outcome assessments through the development of large and effective clinical trials.

a. Operation Brain Trauma Therapy (Lafrenaye et al., 2020)
b. TBI Models Systems (TBIMS) (NDSC, 2016)
c. Common Data Elements (CDE) (NINDS, 2017)
d. Transforming Research and Clinical Knowledge in TBI (TRACK TBI) (TRACK-TBI, 2014)
e. TRACK-TBI Precision Medicine (UCSF, 2018)
f. TBI Endpoints Development (TED) (UCSF, 2014)
g. Collaborative European NeuroTrauma Effectiveness Research in Traumatic Brain Injury (CENTER-TBI) (Maas et al., 2015)
h. Concussion Assessment, Research and Education (CARE) Consortium (CARE Consortium, 2022)

## Cost-Effectiveness of Precision Medicine

Interventions in precision medicine consist mostly of genetic profiling and the detection of predictive biomarkers which can identify patients at risk for a specific disease or a severe variant of a disease. This early identification allows for preventive interventions to reduce the burden of diseases and potentially improve quality of life. In addition, predictive biomarkers can also identify patients who will benefit most from certain therapies. It is estimated that there are over 54,000 diagnostic tests available for over 16,400 genes (NCBI, 2022). Ideally, precision medicine has the potential to reduce costs related to inadequate medical diagnosis and treatment, which ultimately can lead to a better and more effective healthcare system (Shabaruddin et al., 2015).

Precision medicine is a relatively nascent field in medicine. Although evidence regarding cost-effectiveness of its practice is limited, some studies have reported its value. The cost-effectiveness of targeted interventions depends on many factors, such as the prevalence of a certain gene or allele, the accuracy of the test and the costs associated with testing and personalized treatment (Hatz et al., 2014). Since 2014, most economic evaluations of precision medicine have been conducted in the United States and Europe, with a slightly predominance in Europe.

The economics of precision medicine have been largely studied in cancer and cardiovascular related diseases (Kasztura et al., 2019). A recent scoping review concluded that targeted management is at least cost-effective compared to usual care (Kasztura et al., 2019). However, the applied willingness-to- pay thresholds vary widely, from USD 20,000/Quality adjusted life-year (QALY), from UK or Europe studies, to USD 200,000/QALY in USA studies. Meaning, that a targeted intervention considered cost-effective in the USA would not necessarily be considered so in Europe, as the amount of money a society is willing to spend is highly variable (Kasztura et al., 2019). Many factors influence cost-effectiveness, with the lack of quality data being the most commonly cited factor. Some other factors which influence cost-effectiveness are the prevalence of the targeted genetic “condition” in a specific population, costs of genetic testing and associated treatment and the probability of adverse events or mortality (Kasztura et al., 2019). Now, largely known is the fact that TBI represents a massive economic burden for patients, families and society, with annual global costs estimated to be around 400 billion U.S. dollars per the CENTER-TBI (CENTER-TBI, 2022). Although limited evidence exists regards the economics of precision medicine in TBI, it is probably safe to assume that its cost-effectiveness will be of value in the long-term. In conclusion, precision medicine has been increasingly useful for screening, testing and treatment of several diseases (TBI included). However, due to the many factors which influence cost-effectiveness as mentioned above, and the variable thresholds of willingness-to-pay applied, the true cost-effectiveness of precision medicine remains unknown.

## Limitations of Precision Medicine

Despite enormous advances in precision medicine, there are substantial challenges and barriers to overcome before it can be implemented at a major scale in our health care system. Perhaps, one of the largest challenges is the need for significant resources to improve data collection, storage, and integration with electronic medical records. The incorporation of genetic information into routine clinical care will take years if not decades to reach many health care systems. Since TBI is an extremely heterogenous disease where differences in nosology and their associated comorbidities likely contribute to the varying outcomes among civilian and military populations, an important step is the development of common diagnostic and prognostic nomenclature and common diagnostic elements that can be employed among different fields. A common diagnostic system is necessary to determine the medical, psychosocial, and demographic factors influencing prognosis, potentially decreasing the variability in outcomes reported. In addition, widespread dissemination among the population regarding the clinical meaning and value of precision medicine is required. Efforts are needed to secure patient’s engagement and trust. Lastly, patient anxiety, the fear for unnecessary expensive tests and procedures, and privacy concerns might be just a few deterrents to achieve patient participation.

## Conclusion

Over the past two decades, our understanding of the pathophysiology of traumatic brain injury has grown tremendously. However, despite major clinical trials in neuroprotection and neuromonitoring we are still not able to make a significant impact in the long-term outcome of patients who suffer from this disease. This is probably in part due to its complex nature and heterogenicity. It is likely that our current “protocolized” approach to the management and diagnosis of the disease has resulted in our inability to improve outcomes. This calls to a shift towards an individualized approach that should also consider the patient’s biology, environment, genomics and metabolism. Indeed, the integration of all aspects of precision medicine sound like a gigantic challenge that would certainly discourage anyone. But the effective execution of precision medicine in TBI would likely require much more than just the creation of standardized protocols. Probably the development of clinical trials assessing the combined effect of neuromonitoring, genomics, microbiome analysis, etc. would provide us with better tools to fight this devastating condition. Additionally, it is fundamental to standardize phenotype data collection, use standard data elements, and continue to create databases to allow for multicenter research studies combining samples to effectively power genomic research.
